# Ultrasound guided pulsed radiofrequency neuromodulation of sphenopalatine ganglion treatment for allergic rhinitis in childern: A case report

**DOI:** 10.1002/ccr3.9436

**Published:** 2024-09-19

**Authors:** Zeyang Geng, Chenyang Wang, Ruilin Wang, Aimin Zhang

**Affiliations:** ^1^ Pain and Quality of Life Management Center SuperiorMed and Perennial Hospital Chengdu China; ^2^ Department of Pain Management Zhongnan Hospital of Wuhan University Wuhan China; ^3^ Department of Anesthesiology The Affiliated Hospital of Guizhou Medical University Guiyang China; ^4^ Department of Anesthesiology, Sichuan Clinical Research Center for Cancer, Sichuan Cancer Hospital & Institute, Sichuan Cancer Center Affiliated Cancer Hospital of University of Electronic Science and Technology of China Chengdu China

**Keywords:** allergic rhinitis, case report, neuromodulation, pulsed radiofrequency, sphenopalatine ganglion

## Abstract

Allergic rhinitis (AR) is a chronic noninfectious inflammation of the nasal mucosa mediated primarily by allergen‐specific immunoglobulin E (IgE) in atopic individuals after exposure to allergens, with the involvement of non‐IgE‐mediated mechanisms and neuroimmune dysregulation. Conservative treatment of AR is ineffective in children who lack compliance, and traditional surgical procedures are risky, making treatment of this community challenging. The sphenopalatine ganglion (SPG), aka pterygopalatine ganglion, is the largest of the four parasympathetic ganglia located within the head region, existing as a bilateral pair. The fibers that arise from the SPG regulate secretomotor functions and provide sensation from various structures, including the lacrimal glands, the mucous membranes of the oropharynx, nasopharynx, nasal cavity, and upper portion of the oral cavity. Previous studies suggest that SPG plays a much crucial role in the neuro‐related pathophysiological mechanisms of AR. Pulsed radiofrequency (PRF) is a commonly used technique in pain management to produce neuromodulatory effects without damaging nerve tissue. Previous research suggests that SPG dysfunction is one of the important pathophysiological mechanisms of trigeminal autonomic cephalalgia, and PRF targeting SPG can effectively exert neuromodulatory effects to improve its symptoms. We thus predicted that the application of PRF for neuromodulation of SPG would be beneficial for symptom remission in AR. We report the first case of AR successfully treated with PRF targeting the SPG, symptoms did ameliorate during the 24‐week follow‐up period, as manifested by the disappearance of nocturnal open‐mouth breathing and its murmur and a significant reduction in the frequency and severity of daily episodes of nasal congestion, tearing, and conjunctival congestion, which diversifies clinical interventions for AR.

## 1 INTRODUCTION

Allergic rhinitis (AR) is a chronic noninfectious inflammation of the nasal mucosa mediated primarily by allergen‐specific immunoglobulin E (IgE) in atopic individuals after exposure to allergens, with the involvement of non‐IgE‐mediated mechanisms and neuroimmune dysregulation.^1^, ^2^ Typical symptoms of AR include paroxysmal sneezing, clear watery runny nose, itchy nose and nasal congestion. If the causative factor is dominated by indoor allergens (dust mites, cockroaches, animal dander, etc.), the symptoms tend to flare up year‐round, with non‐negligible impact on the patient's quality of life.^3^ When conservative treatment methods do not work in patients with AR, clinician would consider surgical procedures, which are often associated with a considerable risk of complications, e.g. hemorrhage, infection and empty nose syndrome.^3^

Previous clinical studies have shown that acupuncture of the sphenopalatine ganglion (SPG) is effective in relieving the symptoms of AR. However, this kind of treatment needs to be administered multiple times to achieve clinical relief.^4^

Pulsed radiofrequency (PRF) is a commonly used technique in pain management to produce neuromodulatory effects without damaging nerve tissue.^5^, ^6^, ^7^ However, it is not yet known whether PRF can overcome the limitations of conventional treatments for AR. Accordingly, finding a technique that meets the safety and efficacy requirements without the necessity of multiple administrations is deemed necessary for children with AR who fail to respond to conservative treatments. Here, we present first case of clinically relieved AR in a child after a single ultrasound‐guided PRF of bilateral SPG.

## 2 CASE PRESENTATION

### 2.1 Case history and first visit

**FIGURE 1:**
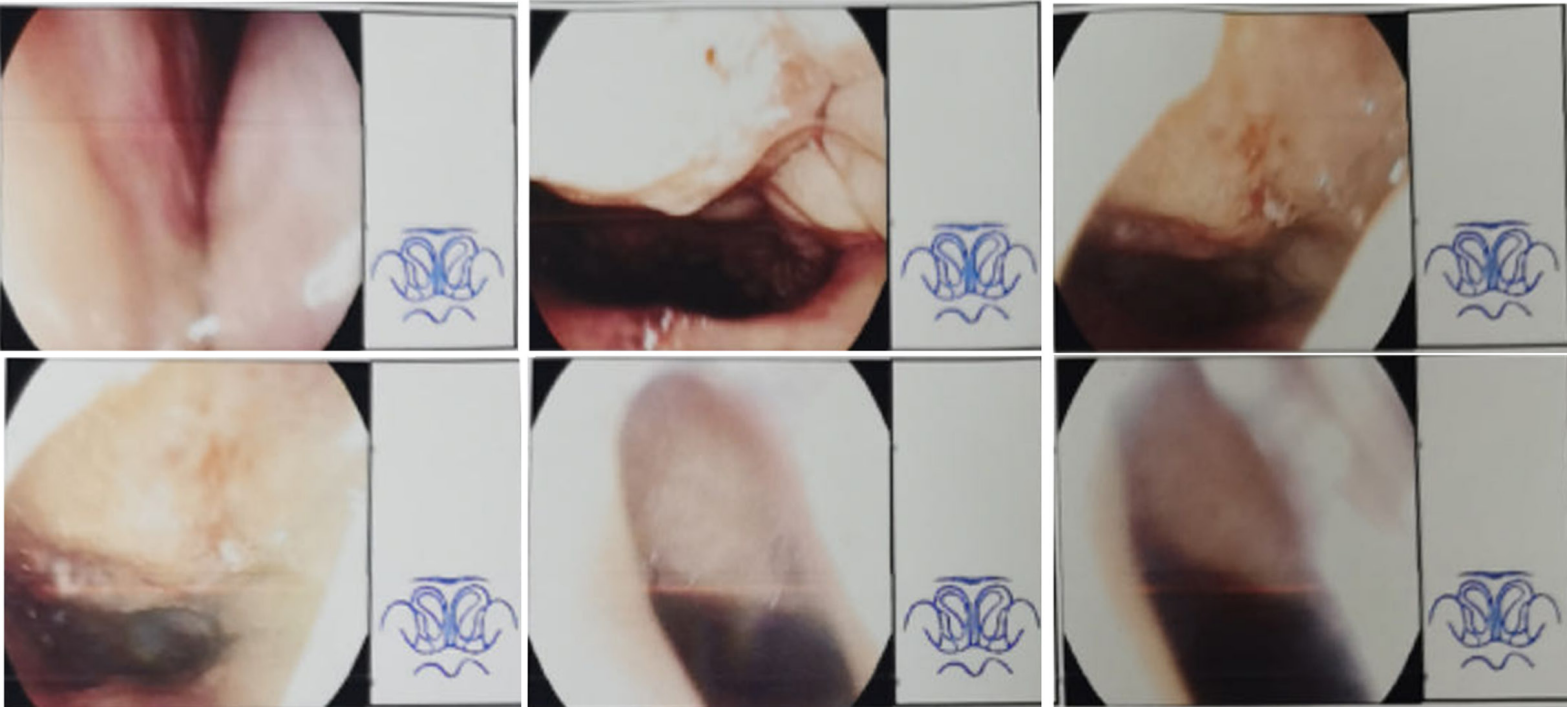
Preoperative fiberoptic nasolaryngoscopic findings of the child in this case. These charts suggested irregular deviation of the nasal septum, bilateral hypertrophy of the middle and inferior turbinates, and adenoid hypertrophy was seen in the nasopharynx, blocking the posterior nasal aperture by about 70%. It was obtained by the child's parents using a smartphone to flip through the original report, and they gave us permission to publish the charts.

A 6‐year‐old female bothered from paroxysmal sneezing, nasal itching, and nasal congestion combined with open mouth breathing during sleep for over 30 months. Electronic nasopharyngoscopy of the child suggested irregular deviation of the nasal septum, bilateral hypertrophy of the middle and inferior turbinates, and adenoid hypertrophy was seen in the nasopharynx, blocking the posterior nasal aperture by about 70% (Figure 1). She also underwent allergy testing (including skin prick test and serum‐specific IgE detection) in local hospital, suggesting that she was extremely sensitive to dust mite and the value of total IgE was 876 IU/mL. Hence she was diagnosed with AR and adenoid hypertrophy. It is noteworthy that the child had frequent previous upper respiratory tract infections with fever and convulsions. The child was already treated with medication, for example, nasal betamethasone, oral montelukast, etc., but did not work well, so surgeon recommended surgery treatment under nasal endoscopy, that is, inferior turbinoplasty, septum correction, pterygoid neurectomy and adenoidectomy. Her parents considered the high risks of surgery and then approached us for help.

Prior to the first visit, the child had completed the basic preoperative investigations as per our request and was fasted from food and drink on the day of the visit. It was made in the outpatient clinic room where a history was taken and the total nasal symptom score (TNSS) was completed with the assistance of her parents. Subsequently, we performed an infrazygomatic arch bilateral pterygopalatine fossa ultrasound evaluation using an ultrasound machine (FUJIFILM SonoSite, Inc, Bothell, USA) with 5–2 MHz transducer.

In terms of differential diagnosis, we excluded vasomotor rhinitis (markedly elevated total IgE), eosinophilia syndrome, infectious rhinitis, pharmacologic rhinitis (strong positive allergen test), and aspirin intolerance syndrome (no history of relevant medication use).

The details of the PRF of SPG procedure and the related potential complications have been fully communicated to the parents of the children, and the most common complication is reversible hematoma of the face, which is caused by accidental injury to the maxillary artery during the puncture process.

At the end of the clinic visit, the child was sent to the post anesthesia recovery unit to establish intravenous access.

### 2.2 Methods: PRF of SPG

The child was transferred to the operating room and placed in supine position, oxygen was administered via anesthesia mask, and cardiac and oxygen saturation monitoring was applied. After sedation was successfully administered, local infiltration anesthesia was applied in the treatment regions of bilateral face.

**FIGURE 2:**
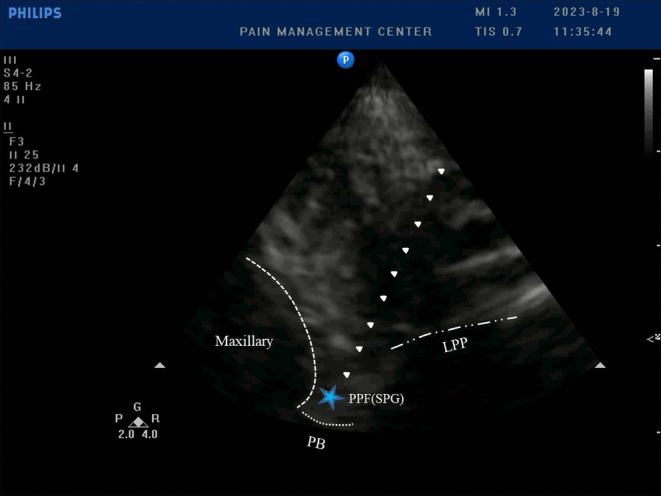
Ultrasound‐guided percutaneous puncture of the SPG by subzygomatic arch approach. After assisting the child to open her mouth, an ultrasound image of the pterygopalatine fossa was visible. The white arrows indicated the trajectory of the radiofrequency cannula and the blue pentagram represented the location of the SPG. PB, palate bone; PPF, pterygopalatine fossa; LPP, lateral pterygoid plate.

Then we confirmed the target of puncture, i.e. SPG, by sweeping the infrazygomatic arch pterygopalatine fossa using a ultrasound machine (PHILPS, USA) with cardiac probe fitted with a sterile condom. After confirming the ultrasound image, we punctured the radiofrequency cannula (inomed Medizintechnik GmbH, Emmendingen, Germany) around the SPG using an out‐of‐plane technique under ultrasound guidance, placed the radiofrequency electrode into the radiofrequency cannula and began PRF therapy (Figure 2). The parameters of the radiofrequency therapeutic instrument (Neo Science Co. Ltd., Beijing, China) were set as follows: temperature 42°C, frequency 2 Hz, pulse width 20 ms, voltage 90 V and time 120 s.

At the end of the procedure, we removed the radiofrequency electrode. A pre‐configured mixture of dexamethasone palmitate injection (Mitsubishi Tanabe Pharma Corporation, Japan) 1 mg, methylcobalamin injection (Eisai Co. Ltd, Shanghai, China) 0.25 mg, and 0.9% NaCl injection was then injected to infiltrate the periphery of the SPG (4 mL totally). The procedures were repeated on the other side to complete the whole treatment process.

### 2.3 Outcome and follow‐up

At the first visit (T_0_), TNSS of the child was 20 points. After PRF treatment, the child's nasal congestion was immediately relieved, and open‐mouth breathing was absent by the end of the night. Her parents gave us feedback on the changes in the child's nasal symptoms using videos taken on their smartphones. We conducted a 24‐week (T_1_‐T_12_) follow‐up, which spanned autumn, winter and the following spring, with bi‐weekly TNSS assessments (Table 1). We were unable to obtain a postoperative electronic nasopharyngoscopy due to concerns of the child's parents about the increased risk of nosocomial infection of the upper respiratory tract. During follow‐up, the child had four episodes of upper respiratory tract infection with fever (common cold with catarrhal rhinitis and Influenza A H1N1, etc.), but none of them resulted in convulsions. Nocturnal nasal congestion with respiratory murmur and open‐mouth breathing reappeared during the upper respiratory tract infections. However, after recovery from these four upper respiratory tract infections, the nasal congestion and open‐mouth breathing still showed significant relief.

**TABLE 1:** TNSS collected from first visit and 24‐week follow‐up.

Time Point	T_0_	T_1_	T_2_	T_3_	T_4_	T_5_	T_6_	T_7_	T_8_	T_9_	T_10_	T_11_	T_12_
TNSS	20	0	2	16	3	18	5	7	17	6	15	7	8

*Note*: This table showed the change in nasal symptom scores from the child's first visit to the end of follow‐up. TNSS, total nasal symptom score.

### 2.4 Conclusion

This case report suggests that PRF of SPG can effectively alleviate the clinical symptoms and improve the quality of life of patient with AR.

## 3 DISCUSSION

At this current stage, the conventional treatment principles of AR include environmental control, medication, immunotherapy, and health education. It is worth noting that glucocorticoids are commonly used in treatment regimens, and the use of this drug in this study was aimed at reducing non‐infectious inflammation associated with puncture as well as minor tissue damage produced during radiofrequency. Several complications could occur with the application of glucocorticoids, among which, clinicians need to be careful to monitor the patient's blood electrolytes during the perioperative period, as hypokalemia may occur.^8^

The SPG, aka pterygopalatine ganglion (PPG), is the largest of the four parasympathetic ganglia located within the head region, existing as a bilateral pair. The SPG is responsible for housing the post‐ganglionic parasympathetic neuronal cell bodies, in addition to acting as a conduit for post‐ganglionic sympathetic and sensory axonal fibers. The fibers that arise from the SPG regulate secretomotor functions and provide sensation from various structures, including the lacrimal glands, the mucous membranes of the oropharynx, nasopharynx, nasal cavity, and upper portion of the oral cavity. A group of headache disorders referred to as trigeminal autonomic cephalalgias (TACs), including cluster headaches, are thought to be influenced by the SPG.^9^, ^10^, ^11^ Also studies showed dysregulation of the SPG plays an integral role in the pathogenesis of AR.^1^, ^2^, ^12^ Therefore, various interventions for SPG have been widely studied in the clinic.

There is strong evidence to support the usage of blockade, radiofrequency, or neurostimulation of the SPG to relieve cluster headaches.^13^, ^14^, ^15^, ^16^ In recent studies, the use of acupuncture to stimulate SPG in the treatment of AR has shown some therapeutic benefits, yet patients need to undergo multiple treatments to achieve significant improvement,^4^, ^17^, ^18^, ^19^, ^20^ which pose a great obstacle to children who lack compliance.

The above studies have shown that blockade, radiofrequency and electrical nerve stimulation, as well as acupuncture produced clinically meaningful neuromodulatory effects on SPG. Considering the risk–benefit ratios, patient compliance, and expense of the above interventions, PRF appears to be more feasible. Yet, there is no relevant clinical guideline or expert consensus to support the application of the above methods for the treatment of AR.

In this case, we achieved satisfactory treatment outcomes with minimal trauma, lower risk, and higher patient compliance. As a supplement to traditional AR treatment options, PRF of SPG has considerable clinical applicability. Additionally, we observed that the children's febrile convulsions also improved after treatment, which increased the potential benefit to the patients.^21^ Nevertheless, the disappearance of convulsions during the follow‐up period may be a coincidence and requires further study.

This report described in detail a case of successfully applying ultrasound‐guided PRF of SPG for a child with AR who had failure to previous conservative treatment. Satisfactory outcome of the treatment was found after 24‐week follow‐up. Yet, the underlying therapeutic mechanism and long‐term efficacy are still unknown. In the future, in‐depth basic research on the mechanism of PRF neuromodulation of SPG is needed, as well as a multicenter randomized controlled trial to further investigate the safety and efficacy of this method is necessary.

## AUTHOR CONTRIBUTIONS

**Zeyang Geng:** Conceptualization; data curation; investigation; methodology; project administration; validation; writing – original draft; writing – review and editing. **Chenyang Wang:** Investigation; methodology; writing – original draft; writing – review and editing. **Ruilin Wang:** Data curation; investigation; resources; writing – review and editing. **Aimin Zhang:** Funding acquisition; investigation; project administration; resources; supervision; writing – review and editing.

## FUNDING INFORMATION

This study was supported by Youth Science Foundation of Natural Science Foundation of Sichuan (Grant No. 2022NSFSC1346) and Medical Research Project in Sichuan Province (Grant No. S21052).

## CONFLICT OF INTEREST STATEMENT

The authors declared no potential conflicts of interest with respect to the research, authorship, and/or publication of this article.

## ETHICS STATEMENT

Our institution does not require ethical approval for reporting individual case. This study was performed in accordance with the Helsinki Declaration of 1964 and its later amendments.

## CONSENT

Written informed consent was obtained from the patient to publish this report in accordance with the journal's patient consent policy.

## AUTHORS' NOTE

The reporting of this study conforms to the Case Report Guidelines (CARE) statement.^22^

## Data Availability

Not available.
